# Supplementary material for: MYB repressors and MBW activation complex collaborate to fine-tune flower coloration in Freesia hybrida
Source: Commun Biol. 2020 Jul 27;3:396. doi: 10.1038/s42003-020-01134-6 (PMC7385123; doi:10.1038/s42003-020-01134-6)
Supplement: Supplementary file 1 — Supplementary Information [file 42003_2020_1134_MOESM1_ESM.pdf]

MYB repressors and MBW activation complex collaborate to fine-tune flower coloration in *Freesia hybrida*

Supplementary Table 1. Information of *FhMYB27* and *FhMYBx* gene

Candidate transcripts	Protein sequence length	Top *Arabidopsis* BLAST match	Top BLAST match excluding *Arabidopsis*	Homology (%)	GenBank number of the homology genes used in Blast
FhMYB27	208 residues	NP_195574.1 myb domain protein 4 *Arabidopsis thaliana*	AFH03064.1 R2R3-MYB transcription factor MYB12 *Epimedium sagittatum*	53[a],59[b]	KF985023
FhMYBx	74 residues	OAP11198.1 CPC *Arabidopsis thaliana*	PREDICTED: transcription factor CPC *Elaeis guineensis*	62[a],81[b]	KF985022

[a]% Similarity to *Arabidopsis thaliana*.

[b]% Similarity to other plant species.

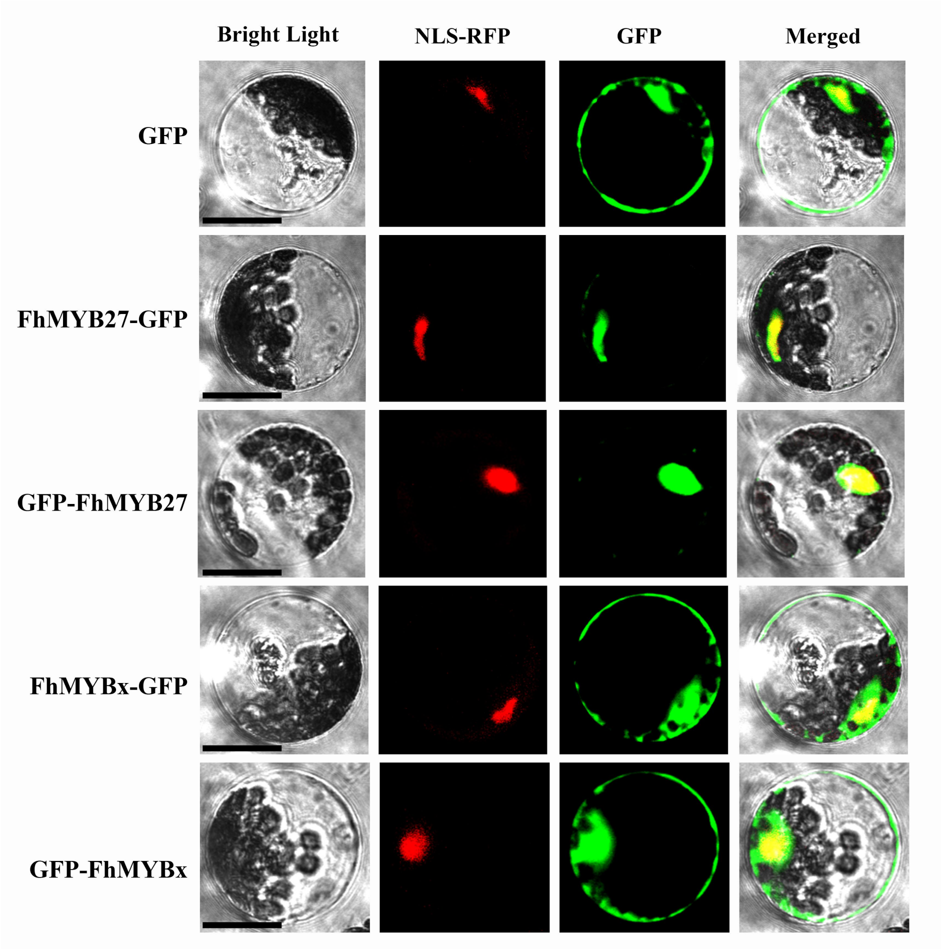

Supplementary Figure 1. Subcellular localizations of FhMYB27 and FhMYBx proteins.

Constructs carrying *GFP*, *FhMYB27-GFP*, *GFP-FhMYB27*, *FhMYBx-GFP* and *GFP-FhMYBx* were transformed into wild type *Arabidopsis* protoplasts. Nuclear localization signal (NLS) - red fluorescent protein (RFP) was included as a nuclear marker. All the proteins were under the control of the CaMV 35S promoter. Results were visualized by fluorescence microscopy after 20-22 h incubation in darkness. From left to right panels in sequence: bright field image, RFP channel, GFP channel, merged image. Bars indicate 25 μm.

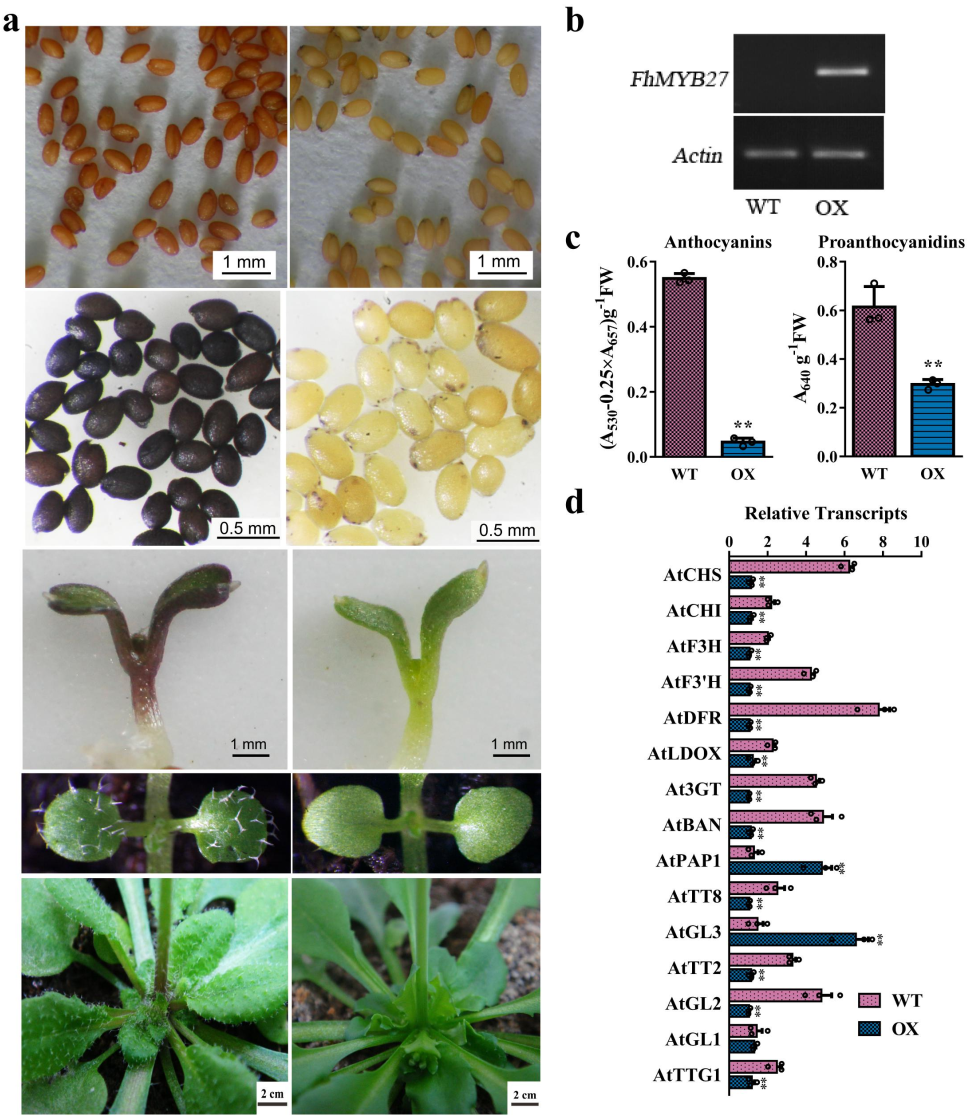

Supplementary Figure 2. FhMYB27 regulated trichome formation, anthocyanin and proanthocyanidin accumulation in *Arabidopsis*.

a Phenotypes of wild type and transgenic *Arabidopsis*. Left panels indicated the wild type tissues or organs, right panels indicated the transgenic tissues or organs. **b** Expression analysis of the *FhMYB27* gene by RT-PCR in the wild type and transgenic lines. **c** Contents of anthocyanins and proanthocyanidins isolated from 4-week old *Arabidopsis* leaves of wild type and *FhMYB27* transgenic lines. **d** Expression analysis of flavonoid biosynthesis and trichome formation related genes by qRT-PCR in the wild type and transgenic lines. Data represented the mean±SD of three replicates. Student's *t*-test was used to analysis the significant difference (*, $p < 0.05$; **$p < 0.01$).

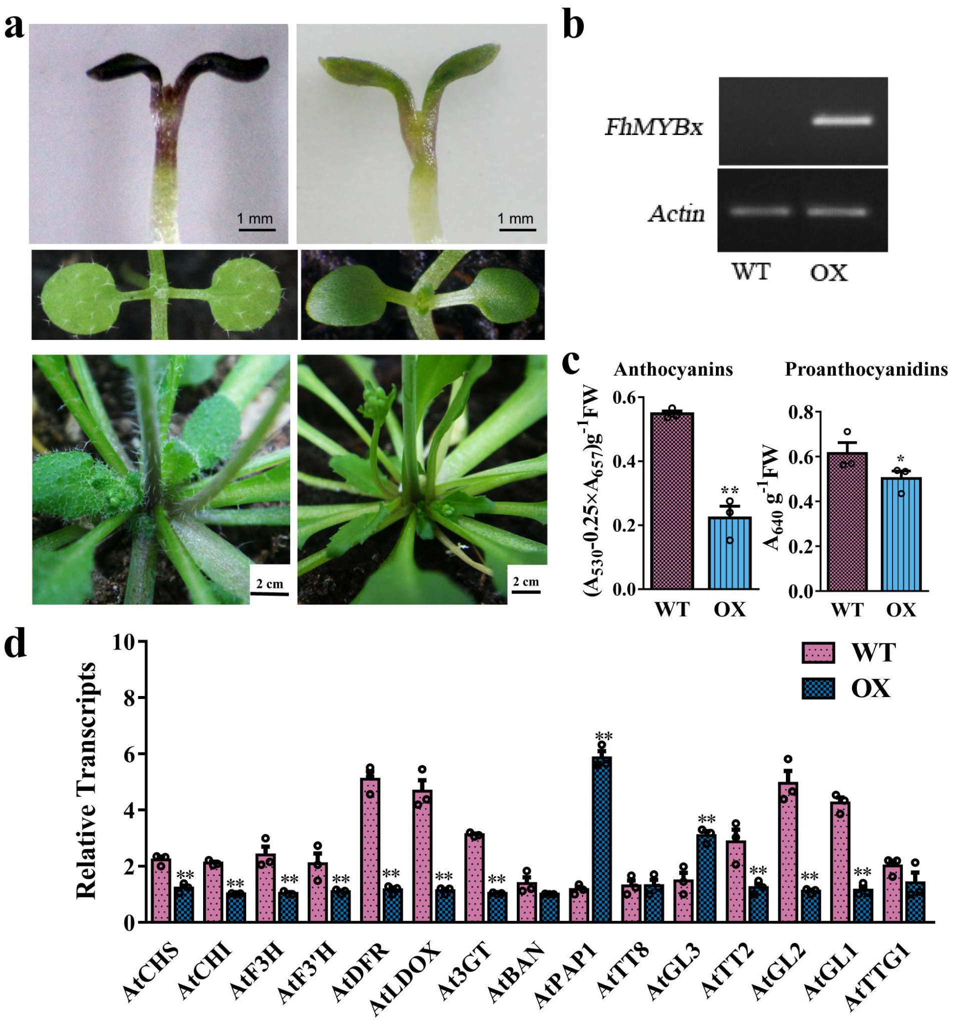

Supplementary Figure 3. FhMYBx regulated trichome formation, anthocyanin and proanthocyanidin accumulation in *Arabidopsis*.

a Phenotypes of wild type and transgenic *Arabidopsis*. Left panel indicated the wild type tissues or organs, right panel indicated the transgenic tissues or organs. **b** Expression analysis of the *FhMYBx* gene by RT-PCR in the wild type and transgenic lines. **c** Contents of anthocyanins and proanthocyanidins isolated from 4-week old *Arabidopsis* leaves of wild type and *FhMYBx* transgenic lines. **d** Expression analysis of flavonoid biosynthesis and trichome formation related genes by qRT-PCR in the wild type and transgenic lines. Data represented the mean±SD of three replicates. Student's *t*-test was used to analysis the significant difference (*, $p < 0.05$; **$p < 0.01$).

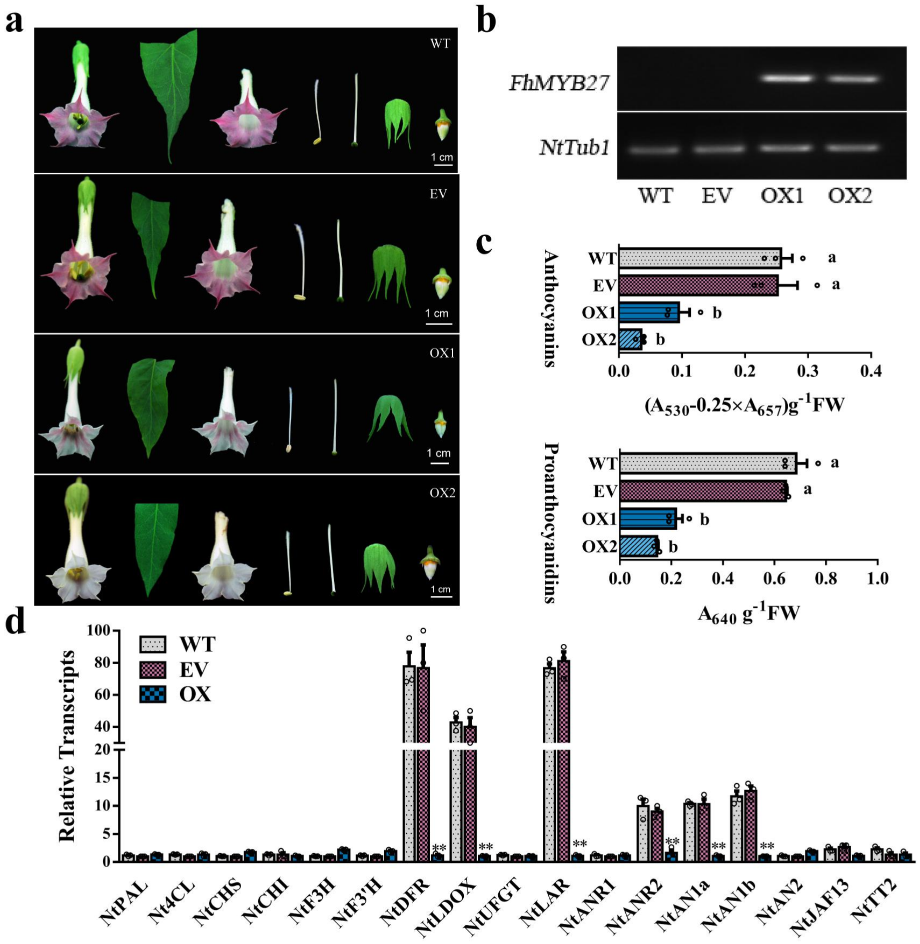

Supplementary Figure 4. FhMYB27 regulated anthocyanin and proanthocyanidin accumulation in tobacco.

a Phenotypes of wild type and transgenic tabacco. WT, wild type tobacco; EV, tobacco expressing empty vector; OX1-2, 2 transgenic lines over-expressing *FhMYB27*. **b** Expression analysis of the *FhMYB27* gene by RT-PCR in the wild type and transgenic lines. **c** Contents of anthocyanins and proanthocyanidins in the wild type and transgenic lines. One-way ANOVA was carried out to compare statistical differences (Duncan's test, $p< 0.05$). **d** Expression analysis of flavonoid biosynthetic genes by qRT-PCR in transgenic lines compared to the wild type plant. As genes in both WT and EV showed high similar expression levels, only the expression results against WT were shown. Data represented the mean±SD of three biological replicates. Sdudent's *t*-test was used to analysis the significant difference (*, $p< 0.05$; **$p < 0.01$).

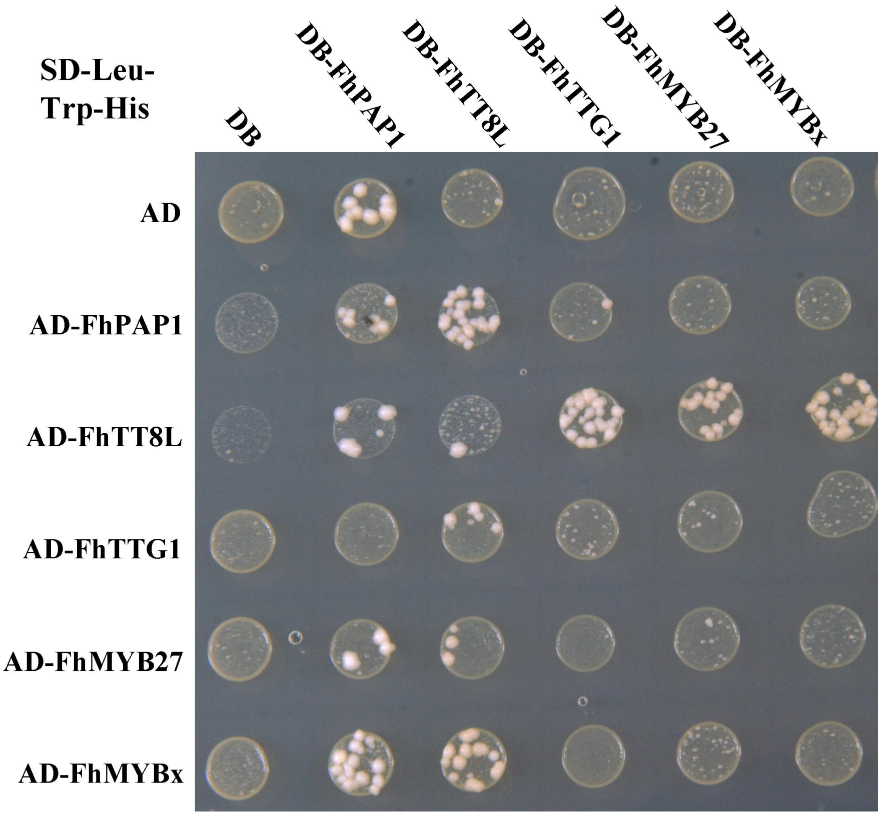

Supplementary Figure 5. Yeast two-hybrid assays demonstrated the direct interactions of *Freesia* proteins.

The ORF of each regulator was fused to the binding domain (DB) or the activation domain (AD) of the shuttle vector. The interaction was tested using the selectable marker, the His auxotroph.

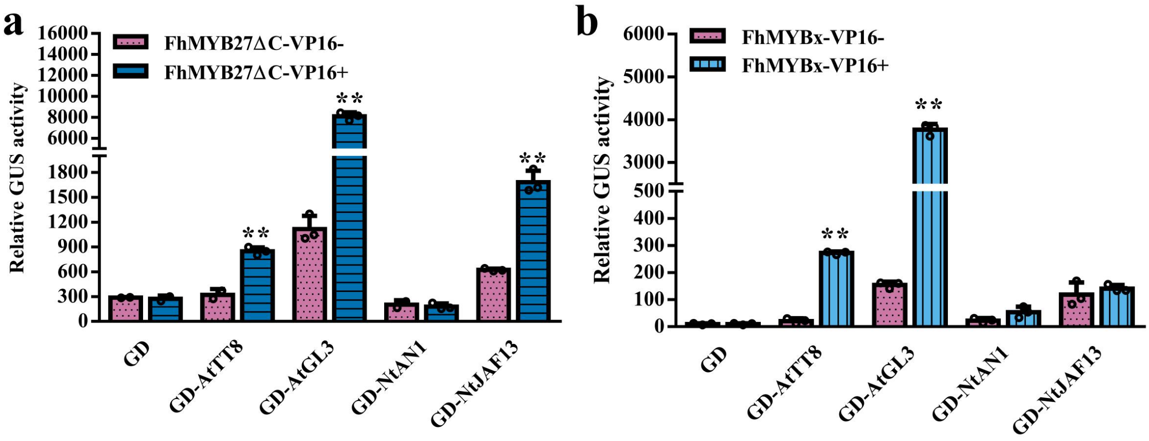

Supplementary Figure 6. FhMYB27 and FhMYBx could interact with different bHLH facors of *Arabidopsis* or tobacco.

a Interactions between FhMYB27 and *Arabidopsis* or tobacco bHLH factors detected by Gal4: GUS system. **b** Interactions between FhMYBx and *Arabidopsis* or tobacco bHLH factors detected by Gal4: GUS system. The reporter and effector constructs were co-transfected into *Arabidopsis* protoplasts. The effector constructs expressed each transcription factor from a *CaMV35S* promoter. FhMYB27ΔC-VP16 (C-terminal repression domain removed + viral activation domain), FhMYBx-VP16 (viral activation domain added). GUS activities were detected after protoplasts incubation for 21 h. Data represented the mean ± SD of three biological replicates. Student's *t*-test was used to analysis the significant difference (*, $p < 0.05$; **$p < 0.01$).

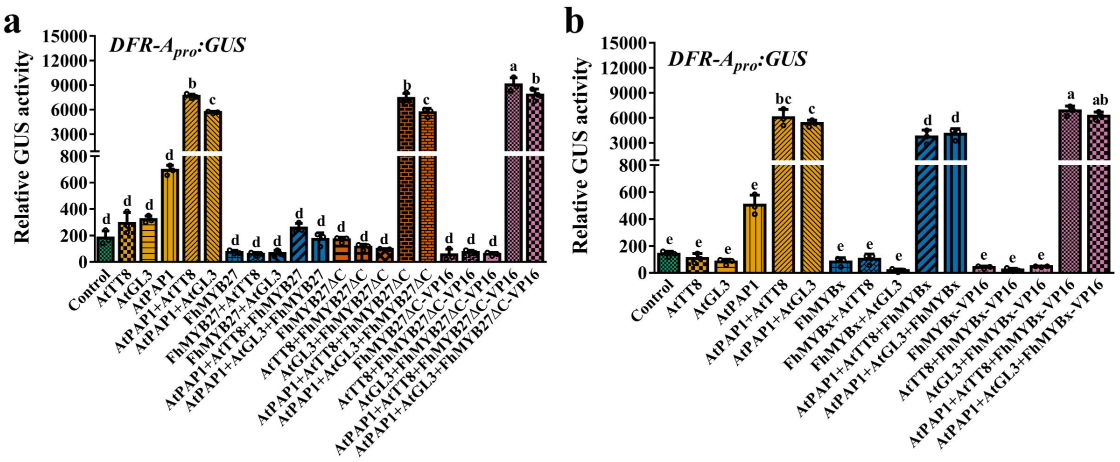

Supplementary Figure 7. FhMYB27 and FhMYBx functioned as corepressors of MBW activation complex to repress anthocyanin accumulation in transgenic *Arabidopsis*.

a Activation and repression assays of the *Arabidopsis DFR* promoter by different combinations of *Arabidopsis* MBW components and FhMYB27. **b** Activation and repression assays of the *Arabidopsis DFR* promoter by different combinations of *Arabidopsis* MBW components and FhMYBx. The reporter and effector constructs were co-transfected into *Arabidopsis* protoplasts. The effector constructs expressed each transcription factor from a *CaMV35S* promoter. Additionally, modified versions of MYB repressors were assayed: FhMYB27ΔC (C-terminal repression domain removed), FhMYB27ΔC-VP16 (C-terminal repression domain removed + viral activation domain), FhMYBx-VP16 (viral activation domain added). GUS activities were detected after protoplasts incubation for 21 h. Data represented the mean ± SD of three biological replicates. One-way ANOVA was carried out to compare statistical differences (Duncan's test, $p < 0.05$).
