# Supplementary material for: MYB repressors and MBW activation complex collaborate to fine-tune flower coloration in Freesia hybrida
Source: Commun Biol. 2020 Jul 27;3:396. doi: 10.1038/s42003-020-01134-6 (PMC7385123; doi:10.1038/s42003-020-01134-6)
Supplement: Supplementary file 4 — Description of Additional Supplementary Files [file 42003_2020_1134_MOESM4_ESM.pdf]

Description of Additional Supplementary Files

File Name: **Supplementary Data 1**
Description: Primers used in this study

File Name: **Supplementary Data 2**
Description: Data underlying graphs
